# Long-Term Outcomes of Elderly Patients Managed Without Early Cholecystectomy After Endoscopic Retrograde Cholangiopancreatography and Sphincterotomy for Choledocholithiasis

**DOI:** 10.7759/cureus.19074

**Published:** 2021-10-27

**Authors:** Kaitlin Edwards, Garrett Johnson, Jessica Bednarz, Krista Hardy, Andrew McKay, Ashley Vergis

**Affiliations:** 1 Surgery, University of Manitoba, Winnipeg, CAN

**Keywords:** cholecystectomy, mortality, elderly, gallstones, choledocolithiasis

## Abstract

Background

Prophylactic cholecystectomy following endoscopic retrograde cholangiopancreatography with sphincterotomy (ERCP-S) remains the gold standard management of choledocholithiasis. Some clinicians propose ERCP-S alone as the definitive management in the elderly, given perioperative complication risks. This retrospective cohort study aimed to assess the long-term efficacy and safety of non-operative management of choledocholithiasis in adults aged ≥70.

Methodology

A total of 252 patients aged ≥70 underwent ERCP from 2004 to 2014 at a single institution. The rates of cholecystectomy, ERCP, complications, and mortality were gathered. Data were linked to a provincial health database to capture follow-up visits to alternate hospitals. Predictors of operation, recurrence, and mortality were analyzed using multivariable regression.

Results

Following ERCP, of the 252 patients, 33 (13.1%) underwent prophylactic cholecystectomy within three months, while 219 (86.9%) were initially managed conservatively. Of the 219 patients, 147 (67.1%) experienced no further choledocholithiasis after conservative management, while 23 (10.5%) patients underwent cholecystectomy. The mean follow-up was 2.9 years. Delayed operative patients were younger (mean age: 77.56 vs. 82.90; p < 0.001) and had lower Charlson Comorbidity Index (CCI) (1.04 vs. 1.84; p = 0.030). When adjusted for age, CCI score, and sex, cholecystectomy was associated with increased survival, with an odds ratio of 0.48 (95% confidence interval = 0.26-0.90; p = 0.021). Perioperative complications occurred in 7/56 (12.5%) patients.

Conclusions

Recurrent choledocholithiasis is common in elderly patients. Despite recurrent symptoms, these patients are unlikely to undergo cholecystectomy. Surgeons operate on patients with greater life expectancy and fewer comorbidities with high success despite advanced patient age. Future prospective studies should examine objective criteria for prophylactic cholecystectomy in this population, given purported safety and benefits.

## Introduction

The incidence of cholelithiasis increases with age, affecting 33% of individuals by age 70 and up to 80% by age 90. An estimated 26% of these patients become symptomatic, requiring medical intervention [1]. When a gallstone enters the common bile duct (CBD), symptomatic choledocholithiasis, cholangitis, and/or pancreatitis can occur. The gold standard management of these conditions includes biliary duct clearance via endoscopic retrograde cholangiopancreatography with sphincterotomy (ERCP-S), followed by prophylactic cholecystectomy to reduce recurrent biliary events [2-5]. Early prophylactic cholecystectomy has typically been defined as occurring within six weeks post-ERCP [3-5].

Elderly patients experience higher perioperative morbidity and mortality rates, given advanced age, comorbid status, and frailty [6]. Therefore, some clinicians have proposed that ERCP-S alone should be considered the definitive management for choledocholithiasis in these patients [7]. This recommendation assumes that a sphincterotomy will prevent additional episodes of CBD stone disease by allowing subsequent stones to pass through the ampulla of Vater, while avoiding the morbidity associated with cholecystectomy. However, in a meta-analysis, the deferral of cholecystectomy in patients with choledocholithiasis of all ages was associated with 2.56 higher odds of death and 5.10-fold higher odds of recurrent biliary pain [8]. While non-operative management is associated with advanced age, there is a paucity of literature examining prophylactic management following choledocholithiasis in the subgroup of patients aged 70 and older. A single randomized trial of 178 patients (age >60 years) who underwent ERCP-S and were randomized to immediate laparoscopic cholecystectomy or expectant management found that recurrent biliary events were significantly more likely in the expectant management group (24% vs. 7%). Moreover, there was a 10% complication rate with no mortalities in the operative cohort [9].

This study aimed to compare the long-term outcomes of patients aged 70 years or older managed operatively versus non-operatively after ERCP-S for choledocholithiasis, cholangitis, or biliary pancreatitis. In addition, it aimed to examine patient factors associated with the decision to operate. An abstract relating to this research was presented at the Canadian Surgery Forum in Montreal, Quebec, Canada, in September 2019.

## Materials and methods

Study design

This is a retrospective cohort study of patients aged 70 or older who underwent ERCP with or without prompt cholecystectomy for the management of choledocholithiasis, cholangitis, and biliary pancreatitis (collectively referred to as CBD stone disease).

Study setting

Data were collected using a chart review of patients at the Health Sciences Centre (HSC), Winnipeg, Manitoba, Canada. The center has a catchment area of 1.5 million people and is the major tertiary care referral center for biliary disease in the region.

Study participants

All patients ≥70 years of age undergoing index ERCP for choledocholithiasis, cholangitis, or biliary pancreatitis from March 2004 to December 2014 at the study site were included. Exclusion criteria included prior cholecystectomy, non-Manitoba resident (unable to link health records to Manitoba Health data), index ERCP outside of the study period, incomplete records, and ERCP for non-gallstone biliary disease (e.g., cancer). The follow-up period was extended until March 2017.

Data sources/variables

Based on the literature, prompt cholecystectomy was defined using a six-week cut-off [3-5]. During analysis, we expanded this cut-off to three months post-ERCP to capture patients purposefully managed with prophylactic cholecystectomy, who would otherwise be misclassified as a failure of conservative management, given long surgical wait times at the study institution, as well as increased preoperative optimization times in an elderly cohort. Demographic information including age, gender, Charlson Comorbidity Index (CCI) score [10], and age-adjusted CCI score were collected from patient charts. The index diagnosis at ERCP, use of sphincterotomy and/or stenting, and hospital admission data were also recorded. The rates and types of post-ERCP complications including pancreatitis, bleeding, and gastrointestinal tract perforation were also captured. Data for subsequent ERCPs were similarly recorded. By linking hospital records to provincial health data through Manitoba Health, additional ERCP and cholecystectomy procedures performed at other centers within the province during the follow-up period were collected. The Manitoba Health Insurance registry provided information about patients lost to follow-up due to relocation or death.

For patients who underwent surgery, information regarding the time from the index ERCP to surgery, surgical acuity, complications, and the length of hospital stay was recorded. The American Society of Anesthesiologists (ASA) score was used as a measure of perioperative health status, and any complications were characterized using the Clavien-Dindo scoring system [11]. Mortality data were collected both from chart review and Manitoba Health Registry data.

Statistical analysis

Student’s t-test and Pearson’s chi-square test were used to determine the differences between patient characteristics in each group for continuous and categorical variables, respectively. Statistical significance was set at a p-value of <0.05. Patients managed conservatively were further stratified by those who went on to have an interval cholecystectomy.

Univariable and multivariable logistic regression models were used to determine the predictors of cholecystectomy following ERCP, as well as predictors of recurrent CBD stone disease. Demographics and index diagnoses with p-values less than 0.20 in the univariable models were added to the multivariable model. Model fit was assessed using the Akaike information criterion and the Bayesian information criterion, where a smaller value indicated a better fitting model. Kaplan-Meier 10-year survival curves were used to analyze mortality rates.

## Results

Patients

**Figure 1:**
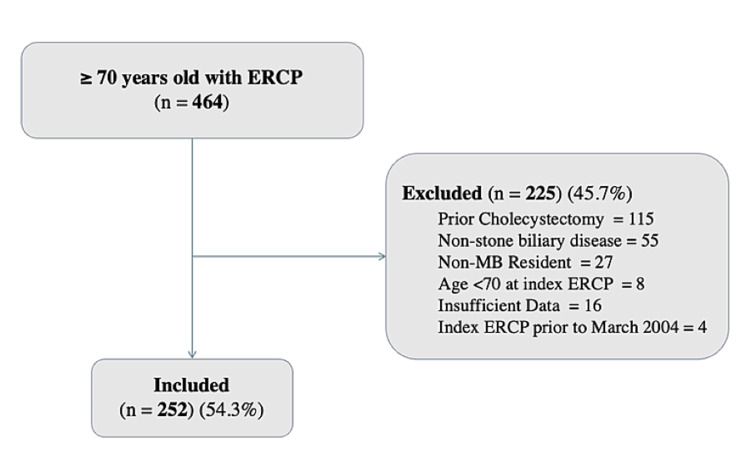
Flowchart illustrating the inclusion process for the retrospective review of 464 patients aged ≥75 who underwent ERCP at the Health Sciences Centre, Winnipeg, Canada, from March 2004 to December 2014. ERCP: endoscopic retrograde cholangiopancreatography; CBD: common bile duct; MB: Manitoba

**Figure 2:**
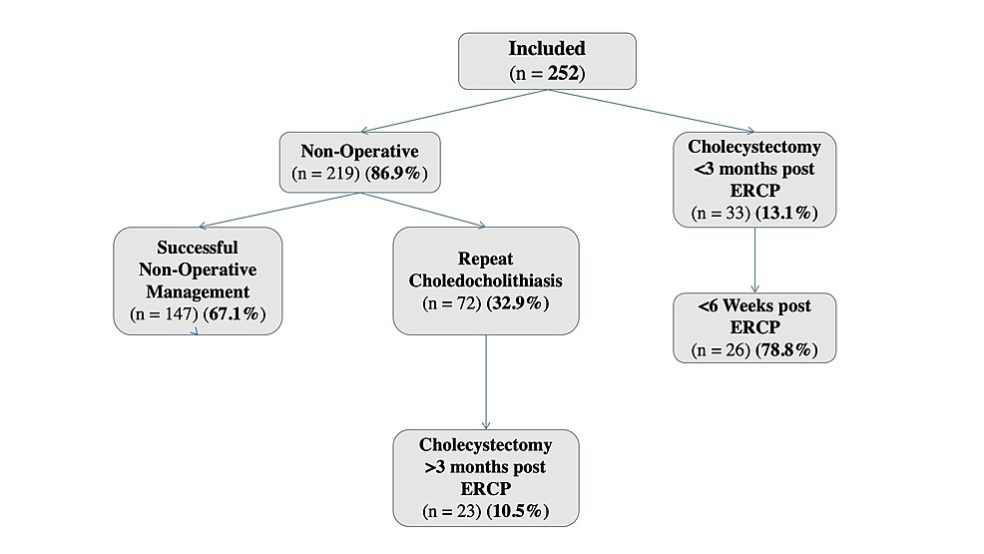
Flowchart illustrating patient treatment assignments and outcomes. ERCP: endoscopic retrograde cholangiopancreatography

A total of 464 patients ≥70 years of age underwent ERCP for CBD stone disease during the study period. In total, 225 participants met the pre-determined exclusion criteria. Finally, 252 patients were included in the study (Figure 1). Overall, 33 (13.1%) patients underwent cholecystectomy within three months following ERCP (Figure 2). The remainder were treated non-operatively. The median and mean follow-up after ERCP for all patients were 2.2 and 2.9 years, respectively.

Predictors of cholecystectomy

Predictors of prophylactic cholecystectomy within three months of CBD stone disease presentation are shown in Table 1. Significant predictors were age (p = 0.019), CCI (p = 0.030), age-adjusted CCI (p = 0.010), cholangitis (p = 0.029), and ASA score ≤3 (p = 0.002). In multivariable analysis, only the age-adjusted CCI score predicted whether a patient would be managed non-operatively, with an odds ratio (OR) of 0.79 (95% confidence interval [CI] = 0.63-0.98; p = 0.029).

**Table 1:** Predictors of prophylactic cholecystectomy within three months of ERCP for CBD stone disease. ASA: American Society of Anesthesiologists; CBD: common bile duct; CCI: Charlson Comorbidity Index; CI: confidence interval; ERCP: endoscopic retrograde cholangiopancreatography; IQR: interquartile range; NA: not applicable; OR: odds ratio; SD: standard deviation All figures show means with the percentage of total in brackets, except where indicated.

	Non-operative (n = 219)	Operative (n = 33)	OR (95% CI)	P-value
Age, years (SD)	82.33 (6.57)	79.53 (4.65)	NA	0.019
Female gender	111 (50.7)	17 (51.5)	1.03 (0.50-2.15)	0.929
CCI (SD)	1.75 (1.66)	1.09 (1.28)	NA	0.030
Age-adjusted CCI (SD)	5.44 (1.94)	4.52 (1.56)	NA	0.010
Choledocolithiasis	139 (63.5)	23 (69.7)	1.32 (0.60-2.92)	0.487
Chalangitis	50 (22.8)	1 (3.0)	0.11 (0.14-0.79)	0.029
Cholecystitis	11 (5.0)	4 (15.2)	2.61 (0.78-8.73)	0.120
Biliary pancreatitis	17 (7.8)	5 (15.2)	2.12 (0.73-6.2)	0.169
Sepsis	7 (3.2)	0 (0.0)	0.42 (0.02-7.58)	0.636
ASA grade ≤3	5 (2.3)	5 (15.2)	7.64 (2.08-28.06)	0.002
Mean follow-up, years (SD)	2.80 (2.36)	3.23 (2.56)	NA	0.336
Sphincterotomy	215 (98.2)	32 (97.0)	0.60 (0.06-5.50)	0.457
Stenting	55 (25.1)	3 (9.1)	0.30 (0.09-1.02)	0.053
Median hospital stay, days (IQR)	6.50 (1.00, 13.00)	3.50 (0.00, 7.00)	NA	0.194

Recurrent common bile duct stone disease

One or more episodes of recurrent CBD stone disease occurred in 72/219 (32.9%) patients who were managed initially with ERCP alone (Figure 2). The average number of ERCPs per person was 1.8. The mean follow-up period was longer in those undergoing secondary ERCP (3.31 vs. 2.45 years; p = 0.018). On univariable analysis (Table 2), the absence of retained stone identified on the index ERCP was the only predictor of recurrent choledocholithiasis, with an OR of 3.84 (95% CI = 1.87-7.88; p < 0.001). No predictors were significant on multivariable analysis. Of the 56 patients who underwent cholecystectomy in both cohorts, 14 (25.0%) had recurrent CBD stone disease after undergoing cholecystectomy.

**Table 2:** Predictors of recurrent choledocholithiasis (univariable analysis). CCI: Charlson Comorbidity Index; CI: confidence interval; ERCP: endoscopic retrograde cholangiopancreatography; NA: not applicable; OR: odds ratio; SD: standard deviation All figures show means with the percentage of total in brackets, except where indicated.

	Recurrence (n = 72)	Success (n = 147)	OR (95% CI)	P-value
CCI score (SD)	1.51 (1.51)	1.86 (1.66)	NA	0.133
Age-adjusted CCI score (SD)	5.13 (2.04)	5.56 (1.89)	NA	0.125
Age (SD)	82.44 (6.25)	82.27 (6.51)	NA	0.854
Male gender	38 (52.8)	70 (47.6)	1.23 (0.70-2.16)	0.473
Choledocholithiasis	44 (61.1)	95 (64.6)	0.86 (0.48-1.54)	0.612
Cholangitis	18 (25.0)	32 (18.6)	1.20 (0.62-2.32)	0.592
Biliary pancreatitis	6 (8.3)	11 (7.5)	1.12 (0.40-3.17)	0.825
Acute cholecystitis	2 (2.8)	9 (6.1)	0.44 (0.09-2.08)	0.299
Sphincterotomy	69 (95.8)	146 (99.3)	0.16 (0.02-1.54)	0.112
No retained stone on index ERCP (%)	23 (31.9)	16 (10.9)	3.84 (1.87-7.88)	<0.001

Predictors of delayed cholecystectomy

On univariable analysis, predictors of cholecystectomy after initial conservative management are shown in Table 3. Significant predictors included young age (mean age: 77.56 vs. 82.90; p < 0.001) and lower CCI score (1.04 vs. 1.84; p = 0.029). Patients were more likely to have had biliary pancreatitis, with an OR of 4.26 (95% CI = 1.35-13.45; p = 0.014). On multivariable analysis, the age-adjusted CCI score was lower in the operative cohort, with an OR of 0.79 (95% CI = 0.634-0.976; p = 0.029). The follow-up time was significantly longer in those conservatively managed who later underwent cholecystectomy at 4.36 years versus 2.62 years (p = 0.001).

**Table 3:** Predictors of cholecystectomy in the non-operative management group (univariable analysis). CCI: Charlson Comorbidity Index; CI: confidence interval; NA: not applicable; SD: standard deviation All figures show means with the percentage of total in brackets, except where indicated.

	Cholecystectomy (n = 23)	No cholecystectomy (n = 196)	OR (95% CI)	P-value
CCI score (SD)	1.04 (1.19)	1.84 (1.69)	NA	0.029
Age	77.56 (5.79)	82.90 (6.44)	NA	<0.001
Females	11 (47.8)	100 (51.0)	0.88 (0.37-2.09)	0.772
Choledocholithiasis	15 (65.2)	124 (63.3)	1.09 (0.44-2.69)	0.854
Cholangitis	1 (4.3)	49 (25.0)	0.13 (0.02-1.04)	0.054
Biliary pancreatitis	5 (21.7)	12 (6.1)	4.26 (1.35-13.45)	0.014
Acute cholecystitis	2 (8.7)	9 (4.6)	1.98 (0.40-9.77)	0.402
Stenting	4 (17.4)	51 (26.0)	0.60 (0.19-1.84)	0.371

Complications

Overall, seven patients required one ERCP after operative management, four patients had two subsequent ERCPs, and three patients had repeat ERCPs three times. Postoperative complications were experienced by 7/56 (12.5%) patients. Complications included urinary retention (1/56), seroma (1/56), wound infections (3/56), and intensive care unit (ICU) admission for failed extubation (2/56). There were no major perioperative bleeds, sepsis, or deaths. The average Clavien-Dindo score was 2.2.

Of the 252 patients, 16 (6.3%) required hospital admission for ERCP-related complications (Table 4). For the seven patients admitted for concerns of perforation, only three were confirmed via imaging. None of the patients required operative management or ICU stay. Six patients required multiple repeat ERCPs for failure to cannulate. There were no documented cases of significant respiratory distress, major cardiac event, or death post-ERCP. Overall complication rates were low in both operative and non-operative groups. Hospital admission at the time of the index ERCP occurred in 93/252 (36.9%) patients. On average, the duration of hospitalization was 16.4 days, with a median of 8.0 days.

**Table 4:** ERCP complications. ERCP: endoscopic retrograde cholangiopancreatography; GI: gastrointestinal

	Non-operative n = 219 (%)	Operative n = 33 (%)
Bleed requiring admission or transfusion	4 (1.8)	0
GI perforation	7 (3.2)	0
Pancreatitis	2 (0.91)	0
Severe respiratory complications	0	0
Sepsis	3 (1.4)	0
Death	0	0
Cardiac events	0	0
Total	16 (7.3)	0

Survival

**Figure 3:**
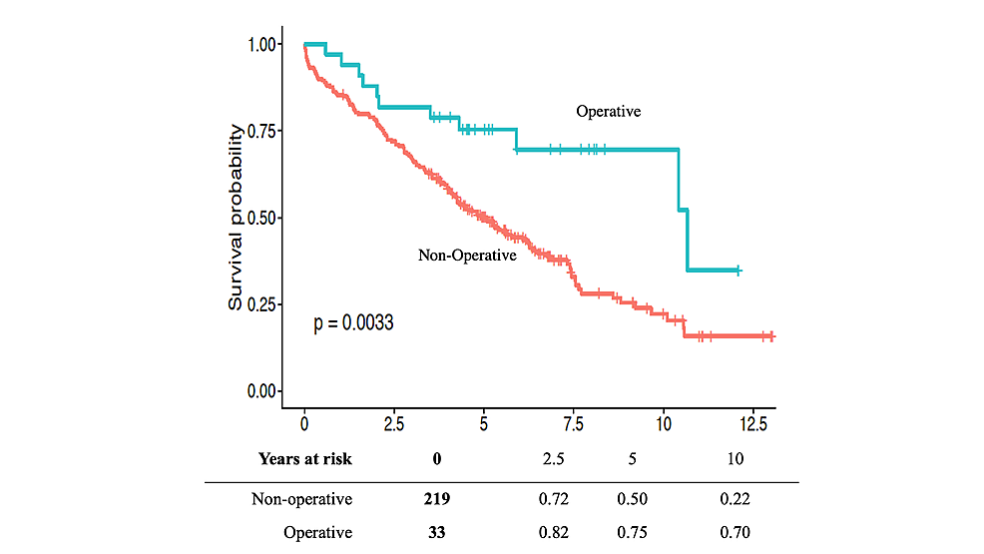
Kaplan-Meier survival curve for patients stratified by prophylactic cholecystectomy within three months of the index ERCP. ERCP: endoscopic retrograde cholangiopancreatography

Kaplan-Meier survival curves demonstrated increased survival in the operative cohort compared to conservative management, beginning shortly after ERCP (Figure 3). Overall, all-cause mortality was lower in the prophylactic cholecystectomy group, 11/33 (33.3%) vs. 139/219 (63.5%) (p = 0.002). Adding delayed cholecystectomies to the operative group increased mortality to 19/56 (33.9%). All cholecystectomy patients had a five-year survival probability of 0.83 compared to 0.45 in the conservative management group, while the 10-year survival probability was 0.60 compared to 0.18 (p < 0.001).

In multivariable analysis, the age-adjusted CCI score was the only significant demographic predictor of mortality, with an OR of 1.24 (95% CI = 1.15-1.35; p < 0.001). When adjusted for age-adjusted CCI score and sex, cholecystectomy within three months was associated with decreased mortality, with an OR of 0.48 (95% CI = 0.26-0.90; p = 0.021). Male sex, while not significant, trended toward higher mortality, with an OR of 1.37 (95% CI = 0.99-1.89; p = 0.06), and was adjusted for on multivariable analysis. Using a more restrictive six-week cholecystectomy cut-off did not reach statistical significance for survival, with an OR of 0.50 (95% CI = 0.24-1.02; p = 0.060).

## Discussion

There was previously limited evidence to guide management in adults aged 70 and older with choledocholithiasis after ERCP-S. A single randomized controlled trial from 2006 examined the role of cholecystectomy in patients ≥60 years old. In the study, recurrent biliary events were significantly more likely in the expectant management group (24% vs. 7%). However, the study was limited in that nearly half of the included patients were younger than 70, and, therefore, had a longer life expectancy than patients one might consider for non-operative management in actual clinical practice [9]. In the present study, we characterized patient outcomes following CBD stone disease in an elderly patient population at a single high-volume Canadian center over a 10-year period. Overall, 32.9% of elderly patients managed initially with ERCP alone for CBD stone disease had recurrent symptoms prompting a visit to the hospital compared to 25.0% of patients following a cholecystectomy. Our findings are consistent with previous studies demonstrating a reduction in recurrent CBD stone disease post-cholecystectomy in younger patients [8].

Complication rates were relatively low in this study, consistent with the frequency noted in prior studies [9,12]. Hospital admission was common and often prolonged, suggesting that while major complications are rare, patient frailty, delayed diagnosis, and interfacility transfers contribute to significant admission duration with associated nosocomial risks.

One argument for forgoing cholecystectomy in those with limited life expectancy is that their likelihood of being affected by their biliary disease is low in comparison to the overall health burden. However, in all-aged adults, early cholecystectomy led to reduced recurrent biliary events within 60 days [13]. Sixty-day follow-up may not be sufficient in identifying CBD stone recurrence as our study demonstrates that many older patients do live long-term (mean survival: 2.9 years). Furthermore, nearly one-third of patients currently selected not to undergo prophylactic cholecystectomy suffer from recurrent symptoms severe enough to pursue hospital treatment. Therefore, careful consideration of risk-reducing cholecystectomy is warranted.

The absence of retained stone on the index ERCP significantly predicted CBD stone disease recurrence. However, as this accounted for less than one-third of total cases, it cannot reliably be used to predict recurrence in clinical practice. Possibly, these patients had more limited sphincterotomies as their duct was easy to clear during the first ERCP, increasing their proclivity for recurrence. It is unclear whether a more aggressive approach to cholecystectomy would be helpful in these patients, given their comorbidities. While laparoscopic cholecystectomy can be considered safe in elderly patients, the literature reports higher laparotomy conversion rates [14]. Postoperative morbidity is correlated to preoperative comorbid status [15], and patients selected for cholecystectomy in the current study had the lowest comorbidity scores. Furthermore, patients with a high comorbid risk may themselves opt out of surgery [9]. Finally, gallbladder removal is not entirely protective against choledocholithiasis in these patients, as evidenced by our high exclusion rate (n = 115) for patients who had a prior cholecystectomy and repeat symptomatic CBD stone disease occurring in 25% of patients who underwent cholecystectomy in our study.

Operative decision-making requires an accurate risk assessment. While the CCI score is validated in predicting 10-year survival [10,16,17], it does not specifically address operative risk. In the present study, ASA grade ≤3 was a significant predictor of operative management on univariable analysis. Previously, Escartín et al. found that in elderly patients undergoing laparoscopic cholecystectomy for acute cholecystitis, ASA grade ≥3 was associated with higher postoperative morbidity and recommended that those with ASA grade 4 should not undergo cholecystectomy [15]. Kenig et al. found that almost all postoperative morbidity experienced by elderly patients post-cholecystectomy was in those with high frailty indices [18]. Frailty has been highly associated with increased perioperative mortality in a meta-analysis [19]. Frailty measures such as the Surgical Apgar score or Geriatric Assessment may be beneficial in future studies on operative management in elderly populations [18,20].

In multivariable analysis, we found a significant difference in mortality between operative and non-operative management utilizing a three-month cut-off. However, interpretation of the observed trend toward improved mortality in the current cohort must be interpreted with caution. This is an observational study, and improved mortality rates likely more closely reflect surgical decision-making rather than inherent mortality benefits from the surgery itself. Consistent with past recommendations, cholecystectomy at this institution is offered selectively to patients with the greatest likelihood of survival and those with fewer comorbidities [8,21]. However, until now, research has not specifically examined the applicability of these recommendations to the very elderly. Our data are consistent with others, demonstrating that elderly patients had lower rates of recurrent CBD stone disease and overall complications if they received surgery [12].

In the present study, a three-month cut-off was used to define early cholecystectomy. Others have used a six-week cut-off [3-5]. Had we used such a short timeframe, very few patients would have been included in the operative group due to inherent delays associated with prolonged surgical waitlists or medical comorbidities. Our data suggest that cholecystectomy within three months likely carries benefit compared to arbitrary four or six-week mandates [12], and may represent a more realistic timeframe for elderly patients who require medical optimization before surgery. The observed trend in this study toward a male mortality preponderance is consistent with past research that indicates males are less willing to accept cholecystectomy, undergo more difficult operations following choledocholithiasis, and have higher mortality rates [8].

This research has some limitations. There is a potential bias inherent to retrospective reviews. Therefore, multivariable analysis was utilized to examine and account for differences between study cohorts. Retrospective data are also subject to unknown confounders, missing variables, and misclassification errors. Medical registry data from outside of the study institution were limited by database information lacking details on emergent versus elective surgery, stenting rates, and etiology for prolonged hospital admissions. Therefore, analysis of these variables is limited to chart review data. One of the study aims was to assess long-term outcomes with patient data collected over a 10-year period. However, the mean follow-up duration was limited to less than three years due to patient deaths, or, in some cases, the index ERCP occurred near the termination of the follow-up period. Finally, this study represents only a single Canadian institution’s experience with this disease over a 10-year period. The applicability of study findings to other institutions and regions is indeterminant and should be confirmed through further research.

Study strengths include its duration and thoroughness of follow-up. We were able to identify all follow-up procedures performed within the province of Manitoba within a 10-year period. The study population is relevant to the general surgeon clinician as cholelithiasis is more prevalent in elderly populations. To our knowledge, this is the first effort to examine an elderly choledocholithiasis cohort for such an extended duration.

## Conclusions

Because many elderly patients with CBD stone disease have recurrent biliary symptoms after ERCP, risk-reducing cholecystectomy should be recommended if comorbidities are not prohibitive. Surgeons seem to appropriately select patients with shorter life expectancies for conservative management in clinical practice. However, many of these patients have recurrent gallstone-related symptoms in their lifetime. Future studies should examine objective risk assessment measures to aid in the decision-making process regarding elderly patients who should be offered prophylactic cholecystectomy, as well as to ensure that symptoms and disease burden in these patients can be minimized.
